# Early-Stage epithelial ovarian cancer: Predictors of survival

**DOI:** 10.1016/j.gore.2022.101083

**Published:** 2022-10-12

**Authors:** Avinash Upadhyay, Vikas Garg, Sandeep Mathur, Prabhat Singh Malik, Neerja Bhatla, Sunesh Kumar, Sachin Khurana, Lalit Kumar

**Affiliations:** a Department of Medical Oncology , All India Institute of Medical Sciences, New Delhi 110029, India; b Department of Gynaecology, All India Institute of Medical Sciences, New Delhi 110029, India; c Department of Pathology

**Keywords:** Early stage, Histological grade, Serous histology, Prognostic factors, Chemotherapy, Fertility sparing surgery

## Abstract

**Background:**

One fifth of patients with epithelial ovarian cancers (EOC) present at an early stage (FIGO stage I & II). However, there is scarcity of literature on the outcomes and its predictors. The aim of the study was to assess relapse free survival (RFS), overall survival (OS) and its predictors in early stage EOC.

**Patients and Methods:**

In this retrospectively study, we included all patients with early-stage EOC diagnosed between January 2010 and December 2018. Patients with synchronous malignancies were excluded. Clinical profile, clinico-pathological characteristics and treatment details were recorded. Patient underwent initial surgery followed by adjuvant chemotherapy in high-risk disease. Patients with stage IC, or stage II or clear cell histology or high-grade histology irrespective of stage/histological subtype were defined as high-risk disease. Fertility sparing surgery (FSS) [unilateral salpingo-oopherectomy with complete surgical staging] was performed in patient willing to preserve fertility. Primary objective was to assess RFS and OS in all patients with early stage EOC. Secondary objectives were to assess RFS and OS in early stage EOC with high-risk disease, predictors of RFS and OS, and outcomes of FSS. Survival probabilities were estimated according to Kaplan-Meier and compared by the log rank test. Cox's regression model was used to analyze the significance of various factors affecting relapse free survival (RFS) and overall survival (OS).

**Results:**

195 patients with early stage EOC were recruited with median age of 47 years (range, 16–80 years). FIGO stage I and stage II were seen in 72 % and 18 % patients respectively. Serous subtype was reported in 58 % and high-grade histology in 66 %. 184 patients (94.0%) underwent optimal staging surgery, including 27 (14%) with fertility sparing surgery (FSS). 133 (91.7 %) of 145 patients with high-risk disease received adjuvant chemotherapy (paclitaxel and carboplatin), while 12 (8.3 %) patients opted to remain on observation. At median follow up of 56 months (95 % CI, 46–64 months), 49 (25 %) patients relapsed [including 3 of 27 (11.1 %) who underwent FSS], 18 patients died of progressive disease, while 31 patients were alive and disease free. Estimated OS at 5 years is 87.6 % (95 % CI 79.9–92.5) and RFS is 73.2 % (95 % CI 64.7–80.0). On multivariate analysis tumor grade was predictive of RFS (HR 2.9, p < 0.04) and OS (HR 9.4, p < 0.02).

**Conclusions:**

This study confirms the excellent outcome for patients with early stage EOC. Histological grade of tumor is a significant predictor of OS and RFS. FSS is feasible in selected patients with early EOC.

## Highlights

• Optimal staging procedure is the initial step.
• Patients with high risk disease should be considered for adjuvant chemotherapy.
• Paclitaxel and carboplatin (AUC 6) every 3 weeks is the standard.
• Tumor grade is predictive of relapse free (RFS) and overall survival (OS).
• 5 year RFS was 73 % and OS was 87 %.

## 1 Introduction

Epithelial Ovarian cancer (EOC) is common gynecological malignancy. Approximately 20 % of patients present with early-stage disease. Following surgery outcome for these patients is very good with five-year survival in the range of 80 % to 93 %, but there is significant risk of relapse in patients with high-risk disease (Berek et al., 2021 Oct, Menon et al., 2021, Torre et al., 2018). As such, role of adjuvant chemotherapy for early EOC has been debated as the earlier trials are flawed by heterogeneous population, inadequate surgery, survival benefit only in certain subsets, use of nonstandard chemotherapy drugs and varying number of chemotherapy cycles.

In International Collaborative Ovarian Neoplasm (ICON) 1 trial, adjuvant chemotherapy demonstrated significant relapse free survival (RFS) and overall survival (OS) advantage. While in Adjuvant ChemoTherapy In Ovarian Neoplasm trial (ACTION) trial, OS benefit was only observed in inadequately staged patients. In both these trials only one third of patients underwent optimal staging surgery and single agent platinum or a combination of cisplatin, adriamycin and cyclophosphamide was administered as adjuvant therapy in majority of patients (not the current standard of chemotherapy). However, combined analysis of these two landmark trials [International Collaborative Ovarian Neoplasm (ICON) 1 and Adjuvant ChemoTherapy In Ovarian Neoplasm trial (ACTION) demonstrated significant benefit of adjuvant chemotherapy in early-stage disease with OS difference of 8 % in favor of chemotherapy (82 % versus 74 %, respectively; P = 0.008). Recurrence-free survival (RFS) was also better for patients in the adjuvant chemotherapy arm (76 % versus 65 %, respectively; P = 0.001) (Colombo et al., 2003, Trimbos et al., 2003, Trimbos et al., 2003 Jan 15). 10 years follow up of ICON1 trial confirmed these results demonstrating benefit of chemotherapy particularly in high-risk disease (Swart, 2007). Furthermore, adequate number of chemotherapy cycles is also debatable. In GOG 157, high-risk early stage EOC were randomized to three or six cycles of paclitaxel and carboplatin. No OS or RFS difference was observed except in patients with serous histology (constituted only 30 % of the trial population), who benefitted from six cycles of adjuvant chemotherapy.

Among important prognostic factors – age, tumor grade, stage, ascites, surface tumor, and tumor capsule rupture have been shown to be independent predictor for relapse in early stage EOC irrespective of adjuvant chemotherapy. The relapse rate varies from 10 % to 50 % in different studies due to heterogeneity of population included. e.g. for FIGO stage IC-II – 5 year OS ranges from 60 to 70 % compared to 90 % for stage IA-IB. (Cannistra, 2004 Dec 9, Tropé and Kaern, 2007 Jul 10, Matz et al., 2017 Feb, Hsieh et al., 2019, Wei et al., 2016 Nov 11, Tognon et al., 2013, Bamias et al., 2011 Oct 1, Lheureux et al., 2019 Mar 23).

With excellent survival, the issue of fertility sparing surgery (FSS) in young EOC patients is an important consideration; many of them are unmarried or have not yet completed family. FSS may be considered especially if they have low risk (stage IA or IB with low grade and non-clear cell histology) EOC. (Schuurman et al., 2021 Feb 28, Liu et al., 2020 Apr 15) Recurrence rate after FSS in early EOC varies from 9.9 % for stage IA/IB, 15.4 % in stage IC to 40 % in stage II. (Kwon et al., 2009 Mar) A recent metanalysis found no difference in survival between FSS and radical surgery in stage- I disease. (Schlaerth et al., 2009 Oct). NCCN guidelines also recommend unilateral salpingo-oopherectomy (USO) or bilateral salpingo-oopherectomy (BSO) with uterine preservation for selected stage IA-IIA patients desiring to preserve fertility. (Armstrong et al., 2022 Sep 1).

In the current study, we report long-term outcome and predictive factors for early stage EOC. We also analysed the role of fertility sparing surgery (FSS) and adjuvant chemotherapy regimen in high risk patients treated uniformly with six cycles of paclitaxel and carboplatin.

### 1.1 Patients and Methods

All women diagnosed and treated for early ovarian cancer at our institution between January 2010 and December 2018 were included in this retrospective study. Patient baseline characteristics, demographic data, clinical features, histopathological profile, serum markers, radiologic findings, stage, operative procedure, and adjuvant treatment were recorded. Histopathological slides were reviewed in all patients to determine the tumor grade. Grading was done as either high grade or low grade on basis of WHO classification (Hohn et al., 2021). Residual disease after surgery was considered in presence of macroscopic pelvic disease after surgery. Inadequate staging surgery defined if complete surgical staging not performed. All patients were staged according to FIGO (Federation Internationale de Gynecologie et d Obstetrique) 2014 classification (Prat et al., 2014). Primary objective was to assess relapse free survival (RFS) and overall survival (OS) in all patients with early stage EOC. Secondary objectives were to assess RFS and OS in early stage EOC with high-risk disease, predictors of RFS and OS, and outcomes of FSS. The Study was approved by the Institute’s Ethics Committee (IECPG-559/26–9-2019, RT-10/24–10-2019).

### 1.2 Risk grouping

Based on our institutional protocol, patients were defined as low risk if they had stage IA or IB with low grade and non-clear cell histology. These were advised close observation after surgery. Patients were defined as high risk if they had high grade tumor irrespective of stage subtype or those with stage IC, or stage II or clear cell histology. These patients were advised adjuvant chemotherapy with six cycles of paclitaxel and carboplatin (AUC 5–6) every 3 weeks. Women age more than 70 years received single agent chemotherapy using carboplatin. Toxicity was recorded as per common terminology criteria for adverse events (CTCAE) 4.0 criteria.

### 1.3 Follow up

Patients were followed up every-three months during first two years, six monthly for next three years and yearly thereafter. On each visit patients underwent detailed physical examination, serum CA125 and ultrasound abdomen. Computed tomography (CT) scan abdomen/pelvis was done, if there was a rising serum CA125 or if patient became symptomatic.

### 1.4 Statistical analysis

Overall survival (OS) was calculated from the date of surgery to the date of death or last date of follow up. Relapse free survival (RFS) was calculated from the date of surgery to the date of relapse or last date of follow up. Survival probabilities were estimated according to the method of Kaplan-Meier and compared by the log rank test. Cox's regression model was used to analyse the significance of various factors affecting RFS and OS. Statistical analysis was carried out using STATA software version 13.0. Data has been censored on 31st December 2020.

## 2 Results

### 2.1 Baseline characteristics

**Fig. 1:**
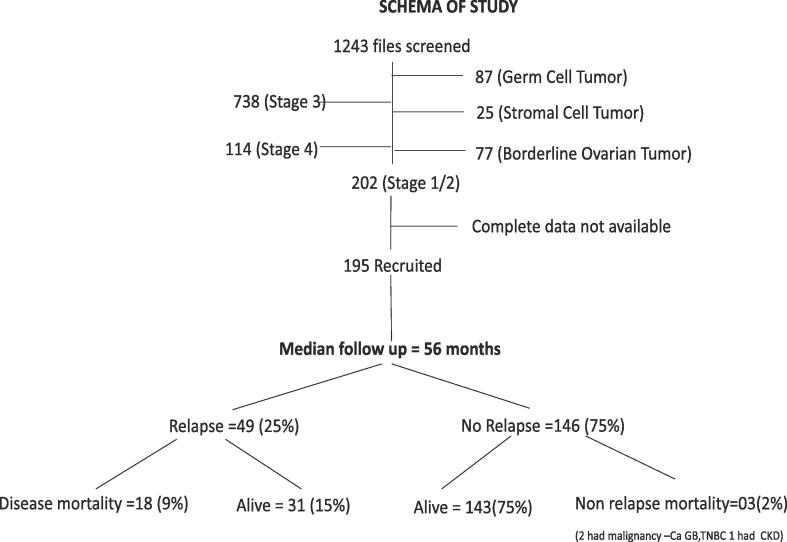
Study schema of the current study.

During the study period, 1243 patients with ovarian cancer were registered, of which 1054 (84.7 %) were epithelial ovarian cancers (Fig. 1). Of these 195 (18.5 %) patients had early-stage EOC and were included in the current study. Baseline characteristics of the study population are given in Table 1 and Table 2. Stage I and II constituted 82 % and 18 % respectively. The median age at presentation was 47 years (range, 16 to 80 years). The most frequent symptom at diagnosis was abdominal pain (66 %) followed by abdominal distension (29 %) and vaginal bleeding (26 %). Serous histology (58 %), endometrioid (17 %) and mucinous (17 %) were common histologic subtypes. Patients with mucinous histology were a decade younger at the time of diagnosis (median age 34 years) as compared to other histologic subtypes. In 65 % of cases, high grade histology was noted, and 71 % of patients had elevated baseline serum CA125 values. The median CA125 values were higher (p = 0.03) in stage II (220 U/ml, interquartile range [IQR] 87–403) compared to stage IA/IB (73 U/ml, IQR 16–184) and stage IC (169 U/ml, IQR 22–535).

**Table 1:** Baseline characteristics of patients with Early Ovarian Cancer.

Baseline Characteristics	n = 195 (%)
Age (in years)	47 (16–80) years
Parity Nulliparous Multiparous with 1 kid Multiparous with greater than 1 kid	41 (21.0 %) 25 (12.8 %) 129 (66.1 %)
Symptom duration (years)	3 (0.1–18) months
Histology Serous Endometrioid Mucinous Clear cell Brenner’s Mixed type	114 (58.4 %) 33 (16.9 %) 33 (16.9 %) 13 (6.6 %) 01 (0.5 %) 01 (0.5 %)
Tumor grade High grade Low grade Not available	117 (60.0 %) 64 (32.8 %) 14 (7.1 %)
Baseline CA125 < 35 U/ml ≥ 35 U/ml Not available	52 (26.6 %) 130 (66.6 %) 13 (06.6 %)

Data is presented as mean (frequency). Age and symptom duration presented as median (range).

**Table 2:** Baseline staging according to FIGO 2014.

Baseline Stage	n = 1054 (%)
**Stage I** Stage IA Stage IB Stage IC Stage IC1 Stage IC2 Stage IC3	**160 (15.1 %)**75 (7.1 %)17 (1.6 %) 21 (1.9 %)30 (2.8 %)17 (1.6 %)
**Stage II** Stage IIA Stage IIB	**35 (3.3 %)**19 (1.8 %)16 (1.5 %)
**Stage III**	**738 (70.0 %)**
**Stage IV**	**114 (10.8 %)**
**Data not available**	07 (0.6 %)

FIGO Federation Internationale de Gynecologie et d Obstetrique.

### 2.2 Treatment

The details of management in EOC in our study have been summarized in Table 3. One hundred eighty-four patients (94.0 %) underwent optimal staging surgery, while FSS was performed in 27 (14 %) patients. Lymph node dissection was not performed (inadequate surgical staging) in three patients (1.5 %), while eight (4.1 %) patients had residual pelvic deposits after surgery. Median duration from date of surgery to start of adjuvant chemotherapy was 46 days (range, 32–62 days). Adjuvant chemotherapy was recommended for 145 patients (74.3 %) with high-risk disease, 133 patients (68 %) received adjuvant therapy, and 12 patients chose to remain under surveillance. Carboplatin and paclitaxel regimen was administered in 128 (96.2%) patients. Most patients (n = 127, 95.4 %) completed all their planned adjuvant chemotherapy cycles. Dose modification was required in only18 (14 %) patients. Most common ≥ grade 3 adverse effects observed during adjuvant chemotherapy were anemia (15.7 %), neutropenia (14.2 %) and thrombocytopenia (9.7 %). All grade neuropathy was seen in 38.3 % patients, while grade 3 or more neuropathy was only present in 3.7 % (n = 5) patients.

**Table 3:** Treatment characteristics of patients with Early Ovarian Cancer.

Treatment characteristics	n = 195 (%)
Type of Surgery Optimal Surgery Residual disease after surgery* Inadequate staging surgery** Fertility sparing surgery***	184 (94.3 %) 08 (4.1 %) 03 (1.5 %) 27 (13.8 %)
Adjuvant therapy Yes Observation No^#^	133 (68.2 %) 50 (25.6 %) 12 (6.1 %)
Chemotherapy (n = 133) Paclitaxel Carboplatin Single agent Carboplatin Adriamycin Cyclophosphamide Cisplatin Cyclophosphamide	128 (96.2 %) 03 (02.2 %) 01 (0.7 %) 01 (0.7 %)
Grade 3 or more toxicity (n = 133) Anemia Neutropenia Thrombocytopenia	21 (15.7 %) 19(14.2 %) 13(9.7 %)

Data is presented as mean (frequency).

*All patients had stage II disease with residual pelvic deposits.

** No lymph node dissection performed (two patients had stage I and one patient with stage II).

*** Unilateral salpingo-oopherectomy with complete surgical staging.

^#^Did not receive adjuvant therapy even though patients were high risk.

### 2.3 Relapse

At a median follow up of 56 months, 49 (25 %) patients had relapsed. All high-risk patients (n = 12) who did not receive adjuvant therapy, three (37.5 %) of eight patients who had residual pelvic deposits post-surgery and two (66.6 %) of three patients who underwent inadequate surgical staging eventually relapsed. Proportion of patient with baseline stage IA/IB, IC and II who relapsed were 18.4 % (n = 17), 27.5 % (n = 68) and 37.1 % (n = 35) respectively. Predominant sites of relapse (Table 4) were peritoneal (48.9 %), retroperitoneal lymph nodes (18.3 %), combined peritoneal and nodal (24.5 %), and distant relapse 4(8.1 %).

**Table 4:** Characteristics at relapse in patients with early EOC.

Characteristics of relapsed patients	n = 49 (%)
Time to relapse (months)	38 (3–96)
Time to relapse <12 months 12–60 months More than 60 months	08(16.3 %) 29(59.1 %) 12(24.5 %)
Stage at baseline IA/IB (N = 92) IC (N = 68) II (N = 35)	17(34.6 %) 19(38.7 %) 13(26.5 %)
Sites of relapse Peritoneal Nodal Peritoneal + Nodal Distant*	24(48.9 %) 09(18.3 %) 12(24.5 %) 04(08.1 %)

Data is presented as mean (frequency). Time to relapse presented as median (range).

EOC Epithelial ovarian cancer * Sites of distant relapse (liver, lung, and brain).

Out of the 49 patients who relapsed, 16 (32.6 %) patients underwent secondary cytoreduction followed by six cycles of chemotherapy (paclitaxel/carboplatin), and rest 33 (67.3 %) received only salvage chemotherapy (Paclitaxel/Carboplatin or Liposomal doxorubicin/Carboplatin). Post salvage therapy complete or partial response was achieved in 42 (85.7 %) patients, while 04 (8.1 %) had stable disease and 03 (6.1 %) had progressive disease. Further lines of chemotherapy were administered in 25 (51.0 %) patients.

### 2.4 Fertility sparing surgery (FSS)

FSS consisted of unilateral salpingo-oophorectomy with preservation of the uterus and contralateral ovary, with staging procedures. Twenty-seven (13.8 %) patients [unmarried (n = 16), nulliparous (n = 8) or family not yet completed (n = 3)] opted for FSS. Median age was 25 years (range, 16 to 37 years). All patients had stage I disease [stage IA −16 (59.2 %), IC- 11 (40.7)], 23 (85.1 %) had low-grade tumors and 15 (56 %) patients had mucinous histology. Adjuvant chemotherapy was advised to 11 patients with stage IC, however, only eight patients received (three patients refused) adjuvant chemotherapy. All three patients who didn’t opt for adjuvant chemotherapy relapsed at 6, 12 and 39 months. Two patients received salvage chemotherapy at relapse and are alive in complete remission at last follow-up, while one patient who refused salvage chemotherapy died of progressive disease. Fertility outcomes were available for six patients, of which two patients successfully delivered five healthy babies.

### 2.5 Survival

**Fig. 2:**
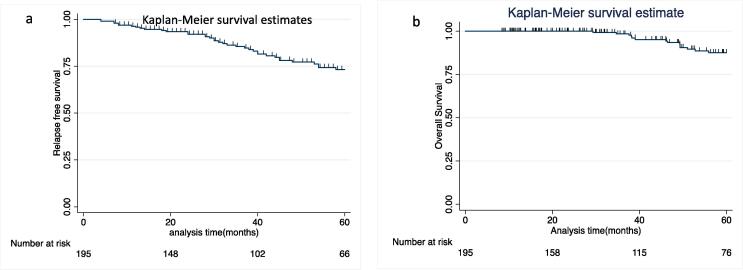
Kaplan-Meier probability of a) relapse free Survival (RFS) and b) overall survival (OS).

**Fig. 3:**
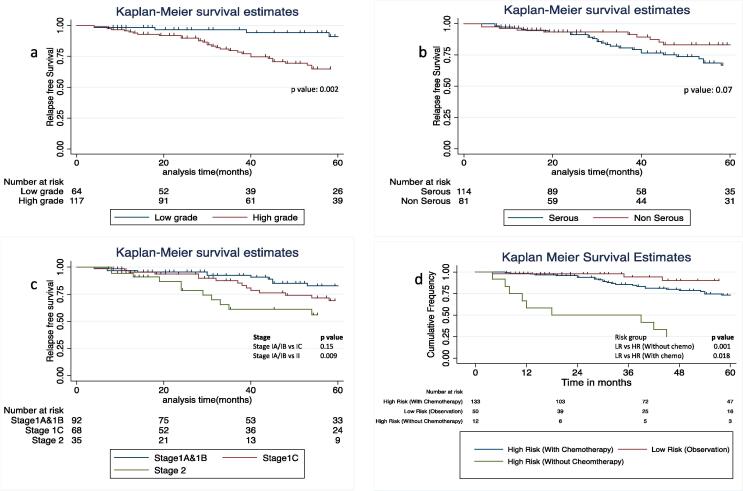
Kaplan-Meier probability of relapse free Survival (RFS) according to a) Grade b) Histologic subtype (serous vs non-serous) c) Stage and d) Risk group.

Currently, 174 (89 %) patients were alive, 18 (9.2 %) patients had died of progressive disease while 03 (1.5 %) patients died of unrelated reasons- one each due to carcinoma gallbladder, triple negative breast cancer and chronic kidney disease. Kaplan-Meier probability of overall survival at 5 years is 87.6 % ± 3.1(95 % CI: 79.9–92.5) for all patients (Fig. 2). On univariate analysis as well as multivariate analysis only grade of the tumor was significant prognostic factor for OS (Table 5). Five-year relapse free survival (RFS) for all patients is 73.2 % ± 3.9 (95 % CI: 64.7–80.0); stage I −77 % and stage II −56 % (Fig. 2). RFS differences according to grade, histologic subtype (serous vs non-serous), stage and risk of relapse (low risk, high risk with chemotherapy and high risk without chemotherapy) has been illustrated in Fig. 3. Five-year RFS in low-risk patients on observation, high-risk patients who received chemotherapy and high-risk patients who didn’t receive chemotherapy was 90.2 % (95 % CI: 71.6–96.9), 73.2 % (95 % CI: 62.7–81.2) and 25.0 % (95 % CI: 6.0–50.4) respectively. On univariate analysis (Table 5) stage, histologic grade, age, and type of surgery (optimal versus residual disease) were found to influence RFS. However, in multivariate analysis only grade of the tumor retained a prognostic role for RFS.

**Table 5:** Predictive variables for relapse free survival (RFS) and overall survival (OS) in early EOC.

Parameters	Univariate analysis		Multivariate analysis	
	HR (CI)	p value	HR (CI)	p value
**Relapse free survival**				
**Stage**	2.0(1.1–3.9)	0.02	1.1 (0.5–2.4)	0.65
**Grade**	3.6(1.6–8.1)	0.002	2.9 (1.1–7.9)	**0.02**
**Age**	2.0 (1.1–3.7)	0.01	1.46 (0.7–2.7)	0.22
**Histological subtype**	1.1(1.0–3.3)	0.07	0.97 (0.4–2.0)	0.93
Surgery type*	2.4(0.9–6.1)	0.06	1.6 (0.5–4.7)	0.38
**Overall survival**				
**Stage**	2.0(0.7–5.2)	0.14	1.3(0.4–3.6)	0.57
**Grade**	11.0(1.4–82.8)	**0.01**	9.4(1.0–83.7)	**0.04**
**Age**	2.0(0.8–4.9)	0.10	1.4(0.5–3.6)	0.45
**Histological subtype**	1.1(0.7–5)	0.20	1.0(0.3–3.0)	0.99
**Surgery type**	0.8(0.1–6.1)	0.85	0.6(0.07–5.2)	0.67

EOC Epithelial ovarian cancer, HR hazard ratio, CI confidence interval, *surgery type (optimal versus sub-optimal) p value < 0.05 was considered significant.

## 3 Discussion

In present study 195 patients with early-stage EOC were included, which constituted 18 % of all ovarian cancers. Median age at presentation was 47 years, which is younger compared to that of western cohort. According to SEER database, the median age of patients with ovarian cancer is 63 years. (Surveillance and Program, 2022). However, multiple retrospective studies have reported younger age (40–50 years) for early stage EOC at diagnosis, similar to present study. (Cannistra, 2004 Dec 9, Tropé and Kaern, 2007 Jul 10, Matz et al., 2017 Feb, Hsieh et al., 2019, Wei et al., 2016 Nov 11). Clinical presentation and median duration of symptoms prior to diagnosis is also alike (Cannistra, 2004 Dec 9, Tropé and Kaern, 2007 Jul 10, Matz et al., 2017 Feb, Hsieh et al., 2019, Wei et al., 2016 Nov 11). In present study 58 % patients had serous histology subtype and two third of all tumors were high-grade. There has been regional variation in the proportion of histologic subtypes with serous histology varying from 14 % in Japan (Hsieh et al., 2019) to 40 % in China (Wei et al., 2016 Nov 11). Other histologic subtypes endometrioid and mucinous were seen in 16.9 % each, while clear cell histology was reported in only 6.6 % patients in present study. In a Japanese study, clear cell histology was most common (37.5 %) followed by endometrioid (27.5 %), serous (14 %) and mucinous (13.3 %). (Wei et al., 2016 Nov 11). Although reasons for such geographic variation in histologic subtypes is unclear, population included in earlier studies was heterogeneous with higher proportion of advanced stage disease. Serum CA 125 was elevated in 71 % of patients in present study which is similar to report by Cuesta et al (de la Cuesta et al., 1999 Jun) but is higher than reported by Tognon et al. (Tognon et al., 2013) Relatively higher frequency of high-grade tumor and elevated CA 125 may be due to over representation of serous histology in present study. (de la Cuesta et al., 1999 Jun). Serum CA 125 values were minimally elevated in stage IA/IB, while values were comparatively higher in stage IC and stage II patients, suggesting a correlation with stage. Therefore, diagnosis of EOC at early stages (stage IA/IB) with higher serum CA 125 requires higher clinical suspicion for higher stage of the disease.

Fertility sparing surgery (FSS) was performed in about one fifth of patients with stage IA and IC disease. Most of these patients had low grade tumor and mucinous histology. Relapses were seen in 11 % of patients (all had high-risk features) who did not receive adjuvant chemotherapy. Similar results were reported by Kwon et al (4.7 %), Schlaerthet et al (15 %) and in a systemic review (15.7 %). (Schuurman et al., 2021 Feb 28, Liu et al., 2020 Apr 15, Kwon et al., 2009 Mar, Schlaerth et al., 2009 Oct) In a recent metanalysis including 2223 patients undergoing FSS, no difference in OS and RFS was observed between FSS and radical surgery. (Liu et al., 2020 Apr 15).

Patients with high-risk features were administered six cycles of adjuvant chemotherapy. Paclitaxel and carboplatin combination remains the preferred chemotherapy regimen for early-stage ovarian cancer. However, the optimal number of cycles of adjuvant chemotherapy remains debatable. (Young, 2003 Jan 15) In GOG157 trial, high-risk patients with early stage EOC were randomized to either three or six cycles of adjuvant chemotherapy (paclitaxel with carboplatin). Although no significant benefit was observed in overall population, patients with serous histology had improvement in survival with additional three cycles of chemotherapy. (Chan et al., 2010 Mar) In current study all high-risk patients who refused adjuvant chemotherapy relapsed, resulting in a worse five-year RFS for these patients than for those who received adjuvant chemotherapy. As most patients were salvaged at relapse, no difference in OS was observed. Due to high relapse rates in patients refusing adjuvant chemotherapy and fact that more than half of patients in our setting have serous histology, use of six cycles of adjuvant chemotherapy is warranted.

At a median follow up of 56 months, five-year RFS was 76.9 % and 55.9 %, while five-year OS was 89.4 % and 78 % for stage I and II, respectively. The results of this study confirm that the prognosis of early-stage EOC is favorable which is consistent with previous studies. (Colombo et al., 2003, Trimbos et al., 2003, Trimbos et al., 2003 Jan 15, Swart, 2007) On univariate analysis, stage, grade, and age were found significant to influence relapse, however only grade of the tumor remained significant on multivariate analysis for both RFS and OS. This is consistent with earlier observations made by Hsieh et al. (2019) and Tognon et al. (2013)), while histologic grade was not a significant predictor for survival in the study by Wei et al. (2016 Nov 11) Most studies (Supplementary Table 1) have reported stage to be significant prognostic factor for RFS as well OS. However, in present study tumor stage lost significance on multivariate analysis. This could be due to a small absolute number of patients. Unlike in study by Hsieh et al. (2019), histology and elevated CA 125 levels were not significant prognostic markers in our study.

Present study includes large data for early-stage EOC in Indian patients with outcome comparable to international studies despite higher frequency of high-risk features. Current study also presented real world evidence of efficacy of six cycles of adjuvant chemotherapy in high-risk early-stage EOC. This will provide greater confidence for use of six cycles especially in high grade and serous histology. Feasibility of FSS in selected patient is further reinforced.

Due to limitation of retrospective design and missing toxicity data, treatment related adverse effects may have been under-reported in the study.

**Conclusion:** Early-stage ovarian cancer constitutes about one fifth of all cases with EOC. Patients present at a younger age and have predominant serous histology with high grade tumors. Long term outcomes are excellent. However, patients with FIGO Stage II and high-grade tumor are at higher risk of relapse and need adjuvant therapy and closer follow up. Histologic grade is an important prognostic factor for relapse and survival. Fertility sparing surgery may be considered in selected stage I patients without impacting survival.

Avinash Upadhyay Data collection, analysis, manuscript draft.

Vikas Garg Manuscript review, patients’ management.

Sandeep Mathur Pathology review.

Prabhat Singh Malik Patients management.

Neerja Bhatla Surgical management.

Sunesh Kumar Surgical management.

Sachin Khurana Patients management.

Lalit Kumar Conceptualization, methodology, manuscript writing, Patients management.

**Authors contributions**.

Funding : None

## Declaration of Competing Interest

The authors declare that they have no known competing financial interests or personal relationships that could have appeared to influence the work reported in this paper.

## References

[b0005] Armstrong D.K., Alvarez R.D., Backes F.J., Bakkum-Gamez J.N., Barroilhet L., Behbakht K., Berchuck A., Chen L.M., Chitiyo V.C., Cristea M., DeRosa M. (2022 Sep 1). NCCN Guidelines® Insights: Ovarian Cancer, Version 3.2022: Featured Updates to the NCCN Guidelines. Journal of the National Comprehensive Cancer Network..

[b0010] Bamias A., Karadimou A., Soupos N., Sotiropoulou M., Zagouri F., Haidopoulos D. (2011 Oct 1). Prognostic factors for early-stage epithelial ovarian cancer, treated with adjuvant carboplatin/paclitaxel chemotherapy: A single institution experience. Gynecol Oncol..

[b0025] Berek J.S., Renz M., Kehoe S., Kumar L., Friedlander M. (2021 Oct). Cancer of the ovary, fallopian tube, and peritoneum: 2021 update. Int J Gynaecol Obstet..

[b0030] Cannistra S.A. (2004 Dec 9). Cancer of the Ovary. N Engl J Med..

[b0035] Chan J.K., Tian C., Fleming G.F., Monk B.J., Herzog T.J., Kapp D.S. (2010 Mar). The potential benefit of 6 vs. 3 cycles of chemotherapy in subsets of women with early-stage high- risk epithelial ovarian cancer: an exploratory analysis of a Gynecologic Oncology Group study. Gynecol Oncol..

[b0040] Colombo N, Guthrie D, Chiari S, Parmar M, Qian W, Swart AM, Torri V, Williams C, Lissoni A, Bonazzi C; International Collaborative Ovarian Neoplasm (ICON) collaborators. International Collaborative Ovarian Neoplasm trial 1: a randomized trial of adjuvant chemotherapy in women with early-stage ovarian cancer. J Natl Cancer Inst. 2003 Jan 15;95(2):125-32. doi: 10.1093/jnci/95.2.125. PMID: 12529345. 10.1093/jnci/95.2.125 12529345

[b0045] de la Cuesta R., Maestro M.L., Solana J., Vidart J.A., Escudero M., Iglesias E. (1999 Jun). Tissue quantification of CA 125 in epithelial ovarian cancer. Int J Biol Markers..

[b9000] Höhn A.K., Brambs C.E., Hiller G.G.R., May D., Schmoeckel E., Horn L.C. (2021). 2020 WHO Classification of Female Genital Tumors. Geburtshilfe Frauenheilkd..

[b0050] Hsieh S.-F., Lau H.-Y., Wu H.-H., Hsu H.-C., Twu N.-F., Cheng W.-F. (2019). Prognostic Factors of Early Stage Epithelial Ovarian Carcinoma. Int J Environ Res Public Health [Internet]..

[b0055] Kwon Y.-S., Hahn H.-S., Kim T.-J., Lee I.-H., Lim K.-T., Lee K.-H. (2009 Mar). Fertility preservation in patients with early epithelial ovarian cancer. J Gynecol Oncol..

[b0060] Lheureux S., Gourley C., Vergote I., Oza A.M. (2019 Mar 23). Epithelial ovarian cancer. Lancet..

[b0065] Liu D., Cai J., Gao A., Wang Z., Cai L. (2020 Apr 15). Fertility sparing surgery vs radical surgery for epithelial ovarian cancer: a meta-analysis of overall survival and disease-free survival. BMC Cancer..

[b0070] Matz M., Coleman M.P., Sant M., Chirlaque M.D., Visser O., Gore M. (2017 Feb). The Histology of Ovarian Cancer: Worldwide Distribution and Implications for International Survival Comparisons (CONCORD-2). Gynecol Oncol..

[b0075] Menon U., Gentry-Maharaj A., Burnell M., Singh N., Ryan A., Karpinskyj C., Carlino G., Taylor J., Massingham S.K., Raikou M., Kalsi J.K., Woolas R., Manchanda R., Arora R., Casey L., Dawnay A., Dobbs S., Leeson S., Mould T., Seif M.W., Sharma A., Williamson K., Liu Y., Fallowfield L., McGuire A.J., Campbell S., Skates S.J., Jacobs I.J., Parmar M. (2021). Ovarian cancer population screening and mortality after long-term follow-up in the UK Collaborative Trial of Ovarian Cancer Screening (UKCTOCS): a randomised controlled trial. The Lancet..

[b0080] Prat J; FIGO Committee on Gynecologic Oncology. Staging classification for cancer of the ovary, fallopian tube, and peritoneum. Int J Gynaecol Obstet. 2014 Jan;124(1):1-5. doi: 10.1016/j.ijgo.2013.10.001. Epub 2013 Oct 22. PMID: 24219974. 10.1016/j.ijgo.2013.10.001 24219974

[b0090] Schlaerth A.C., Chi D.S., Poynor E.A., Barakat R.R., Brown C.L. (2009 Oct). Long-term survival after fertility-sparing surgery for epithelial ovarian cancer. Int J Gynecol Cancer Off J Int Gynecol Cancer Soc..

[b0095] Schuurman T., Zilver S., Samuels S., Schats W., Amant F., van Trommel N. (2021 Feb 28). Fertility-Sparing Surgery in Gynecologic Cancer: A Systematic Review. Cancers..

[b0100] Surveillance, Epidemiology, and End Results Program https://seer.cancer.gov accessed on 16^th^ July 2022.

[b0105] Swart A.C. (2007). Long-term follow-up of women enrolled in a randomized trial of adjuvant chemotherapy for early stage ovarian cancer (ICON1). J Clin Oncol..

[b0110] Tognon G, Carnazza M, Ragnoli M, Calza S, Ferrari F, Gambino A, et al. Prognostic factors in early-stage ovarian cancer [Internet]. 2013 [cited 2021 Feb 15]. Available from: http://ecancer.org/en/journal/article/325-prognostic-factors-in-early-stage-ovarian-cancer. 10.3332/ecancer.2013.325 PMC3680229 23781280

[b0115] Torre L.A., Trabert B., DeSantis C.E., Miller K.D., Samimi G., Runowicz C.D., Gaudet M.M., Jemal A., Siegel R.L. (2018). Ovarian Cancer Statistics, 2018. CA Cancer J Clin..

[b0120] Trimbos J.B., Vergote I., Bolis G., Vermorken J.B., Mangioni C., Madronal C., Franchi M., Tateo S., Zanetta G., Scarfone G., Giurgea L., Timmers P., Coens C., Pecorelli S. (2003). Impact of Adjuvant Chemotherapy and Surgical Staging in Early-Stage Ovarian Carcinoma: European Organisation for Research and Treatment of Cancer-Adjuvant ChemoTherapy in Ovarian Neoplasm Trial. JNCI Journal of the National Cancer Institute.

[b0125] Trimbos J.B., Parmar M., Vergote I., Guthrie D., Bolis G., Colombo N. (2003 Jan 15). International Collaborative Ovarian Neoplasm trial 1 and Adjuvant ChemoTherapy In Ovarian Neoplasm trial: two parallel randomized phase III trials of adjuvant chemotherapy in patients with early-stage ovarian carcinoma. J Natl Cancer Inst..

[b0130] Tropé C., Kaern J. (2007 Jul 10). Adjuvant chemotherapy for early-stage ovarian cancer: review of the literature. J Clin Oncol Off J Am Soc Clin Oncol..

[b0135] Wei W., Li N., Sun Y., Li B., Xu L., Wu L. (2016 Nov 11). Clinical outcome and prognostic factors of patients with early-stage epithelial ovarian cancer. Oncotarget..

[b0140] Young R.C. (2003 Jan 15). Early-stage ovarian cancer: to treat or not to treat. J Natl Cancer Inst..

