# Supplementary material for: Early-Stage epithelial ovarian cancer: Predictors of survival
Source: Gynecol Oncol Rep. 2022 Oct 12;44:101083. doi: 10.1016/j.gore.2022.101083 (PMC9583112; doi:10.1016/j.gore.2022.101083)
Supplement: Supplementary data 1 [file mmc1.docx]

**Supplementary Table-1: Prognostic factors for overall survival in early EOC**

| **Factors** | **Hseih et al(15)**  **HR (p value)** | **Tognon et al(17)**  **HR (p value)** | **Wei et al(16)**  **HR (p value)** | **Present study**  **HR (p value)** |
| --- | --- | --- | --- | --- |
| Stage | 5.97 (0.007) | 7.4 (0.000) | 4.0 (0.01) | 1.3 (0.57) |
| Grade | 2.7 (0.02) | 8.3 (0.001) | 1.0 (0.93) | 9.4 (0.04) |
| Histology | NA | 0.95 (0.80) | NA | 1.0 (0.99) |
| Age | 0.91 (0.73) | 2.3 (0.008) | NA | 1.4 (0.45) |
| CA 125 | NA | 1.5 (0.30) | NA | 1.06 (0.86) |

EOC Epithelial ovarian cancer

HR hazard ratio. p value < 0.05 was considered significant. NA- not available
